# Shotgun Metagenomic Analyses of Microbial Assemblages in the Aquatic Ecosystem of Winam Gulf of Lake Victoria, Kenya Reveals Multiclass Pollution

**DOI:** 10.1155/2023/3724531

**Published:** 2023-07-21

**Authors:** Sandra Khatiebi, Kelvin Kiprotich, Zedekiah Onyando, Clabe Wekesa, Celestine N. Chi, Chrispinus Mulambalah, Patrick Okoth

**Affiliations:** ^1^Department of Biological Sciences, School of Natural Science, Masinde Muliro University of Science and Technology, P.O. Box 190, 50100 Kakamega, Kenya; ^2^Department of Medical Biochemistry and Microbiology, University of Uppsala, P.O. Box 582, 75123 Uppsala, Sweden; ^3^Department of Medical Microbiology & Parasitology, School of Medicine, Moi University, P.O. Box 4606, 30100 Eldoret, Kenya

## Abstract

Lake Victoria, the second-largest freshwater lake in the world, provides an important source of food and income, particularly fish for both domestic consumption and for export market. In recent years, Lake Victoria has suffered massive pollution from both industrial and wastewater discharge. Microplastic biomes, pharmaceutical residues, drugs of abuse, heavy metals, agrochemicals, and personal care products are ubiquitous in the aquatic ecosystem of Winam Gulf. These pollutants are known to alter microbial assemblages in aquatic ecosystems with far-reaching ramification including a calamitous consequence to human health. Indeed, some of these pollutants have been associated with human cancers and antimicrobial resistance. There is a paucity of data on the microbial profiles of this important but heavily polluted aquatic ecosystem. The current study sought to investigate the metagenomic profiles of microbial assemblages in the Winam Gulf ecosystem. Water and sediment samples were collected from several locations within the study sites. Total genomic DNA pooled from all sampling sites was extracted and analyzed by whole-genome shotgun sequencing. Analyses revealed three major kingdoms: bacteria, archaea and eukaryotes belonging to 3 phyla, 13 classes, 14 families, 9 orders, 14 genera, and 10 species. *Proteobacteria*, *Betaproteobacteria*, *Comamonadaceae*, *Burkholdariales*, and *Arcobacter* were the dominated phyla, class, family, order, genera, and species, respectively. The Kyoto Encyclopedia of Genes and Genomes indicated the highest number of genes involved in metabolism. The presence of carbohydrate metabolism genes and enzymes was used to infer organic pollutions from sewage and agricultural runoffs. Similarly, the presence of xylene and nutrotoluene degradation genes and enzyme was used to infer industrial pollution into the lake. Drug metabolism genes lend credence to the possibility of pharmaceutical pollutants in water. Taken together, there is a clear indication of massive pollution. In addition, carbohydrate-active enzymes were the most abundant and included genes in glycoside hydrolases. Shotgun metagenomic analyses conveyed an understanding of the microbial communities of the massively polluted aquatic ecosystem of Winam Gulf, Lake Vicoria, Kenya. The current study documents the presence of multiclass pollutants in Lake Victoria and reveals information that might be useful for a potential bioremediation strategy using the native microbial communities.

## 1. Introduction

Pollution related to freshwater ecosystems is driven by anthropogenic activities known to alter natural biogeophysical processes through increase of eutrophication, acidification, and the input of toxic pollutants [1, 2]. Changes in catchment land-use and riparian vegetation, coupled with downstream sedimentation, nutrient loading, and siltation of both organic and inorganic materials have negatively affected water quality variables and lake biodiversity [3]. The cumulative effects of anthropogenic activities influence ecosystem productivity, species composition, and the genetic diversity of aquatic flora and fauna [4]. In addition, anthropogenic activities lead to massive biodiversity dysfunction and the alteration of microbial community structures and functions [5]. Multiclass pollutants including microplastics, pharmaceutical residues, heavy metals, personal care products, agrochemicals, and drugs of abuse are known to alter microbial assemblages in aquatic ecosystem, and their increased concentration may have deleterious consequences [6]. Freshwater ecosystems are a powerhouse of biodiversity threatened by environmental perturbations including pollution [7]. Ecotoxicological studies indicate that pollution affects aquatic microbes at different organizational and functional levels, including genes, cellular process, and general microbial community responses to polluted ecosystems [8]. Some pathogenic bacterial species are known to cause cancer in humans and other animals through two mechanisms: the production of carcinogenic metabolites and the induction of chronic inflammations [9]. For instance, *Helicobacter pylori* and *Campylobacter jejuni* are reported to cause cancer by induction of chronic inflammation [10]. Several species of *Fusobactrrium* cause colon adenomas as a potential precursor to colon cancer [11]. While *Bacteriodes* produce strong fecal mutagenic compounds, fecapentaenes are associated with human cancers [12].

Pharmaceuticals are found in large quantities in sewage and waste water treatment plants and are increasingly polluting terrestrial, freshwater, and marine ecosystems [13]. Aquatic pollution due to pharmaceuticals including antibiotics is known to lead to antibiotic resistance in natural microbial assemblages, and several species of bacteria found in such a polluted environment have been reported to harbor antibiotic resistance genes [14]. Species of *Pseudomonas*, *Acinobacter*, and *Burkhoideria* isolated from polluted aquatic environment are reported to show multiple antibiotic resistance [15]. Regardless of the negative impacts of microbial pollutants, microbes play key roles in freshwater ecosystem, mediating a large role in vital biogeochemical activities and having a large impact on aquatic community structures [16]. Processes including cycling of nutrients, biodegradation, and neutralization of toxins, among other biogeochemical activities, enhance the flow of matter and energy in aquatic ecosystem [17].

The desire for a better understanding and analyses of changes in polluted aquatic ecosystems has led to the design and application of new technologies like metagenomics [18]. In order to modernize microbiome investigations of microbial processes in contaminated aquatic ecosystems, next-generation sequencing (NGS) technology developments have tended to focus on 16S rRNA. It has been widely employed to decipher the functional role of microbes in the transformation, degradation, and detoxification of dangerous chemicals in contaminated environments [19]. Shotgun metagenomics is an advanced method for quantitative characterization of microbial profiles in different habitats including polluted aquatic ecosystems [20]. Metagenomics is commonly used to generate a large quantity and qualitative information needed to explore the potential roles of the microbial world including detailed functional analyses of native bacterial profiles surviving in multiclass polluted aquatic ecosystems [21]. Such information is important in the management, monitoring, and restoration of polluted aquatic ecosystems [22].

Currently, the spatial and temporal distribution of aquatic microbial assemblages in relation to pollution levels in offshore and inshore ecosystems of Lake Victoria, one of the largest freshwater lakes in the world, remains scanty and poorly documented. Therefore, the purpose of this study was to evaluate the microbial assemblages of the polluted aquatic ecosystem of Winam Gulf in Kisumu, located in the north-eastern part of Lake Victoria, using shotgun metagenomics analyses. This approach has detected microbes across all domains of life and has overcome the bias of the PCR choices used in marker gene sequencing [23]. The in-depth taxonomy, biodiversity, and potential functional analyses of the microbial communities have revealed levels of pollutants that need to be addressed in order to help inform policymakers on the way forward regarding the health of people living along Lake Victoria region.

## 2. Materials and Methods

### 2.1. Study Area

**Figure 1:**
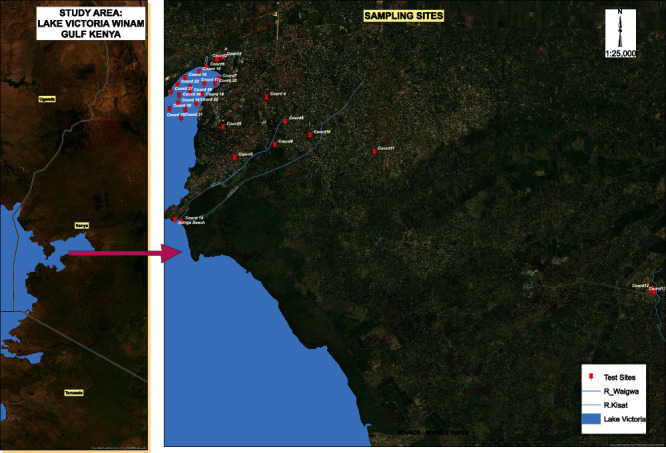
Location of Winam Gulf in Lake Victoria, Kenya, and GPS location of the sampling sites.

Winam Gulf (Figure 1) is an extension of the Northeastern part of Lake Victoria and Western Kenya bordering Uganda [24]. It extends into Kisumu, Homabay, Migori, Busia, and Siaya counties. It is the shallow inlet, 35 mi (56 km long and 15 mi wide), and connected to the main lake by a channel 3 mi. The Winam Gulf lies on latitude 0°14′14.40^″^N and longitude 34° 34′28.79^″^E and experiences annual precipitation from 1000 mm around the lake shores to more than 1800 mm in higher elevations in the eastern areas. The average rainfall of the area is around 1966 mm while the average temperature is 23.1°C per year (Kisumu weather & climate | temperature & weather by month—Climate-Data.Org, n.d.). The area is estimated to hold a population of 397,957 according to the Kenya Population and Housing Census 2019 (KPHC) and is home to small-scale agricultural retail markets, fishing and small-scale industries like tourism, food processing, oil refining, plastics, furniture, and cement. It has also been characterized with several commercial outlets such as supermarkets, educational facilities with a high student population such as RIAT, Kisumu National Polytechnic, and Maseno University, and great lakes. Other major establishments include health and research facilities such as the Kenya Medical Research Institute (KEMRI), Jaramogi Oginga Odinga Teaching and County Referral Hospital, and several privately owned hospitals and clinics.

### 2.2. Study Sites

Sampling was carried out at the flood plains of the inlet rivers, the Kisumu wastewater treatment plant's (WWTP) effluent discharge into the lake, Kisumu industrial effluent, fish landing beaches, storm water entrance points, the Kisumu Water and Sewerage Company (KIWASCO) treatment facility, rivers Kisat, Wigwa, Nyamasaria, Nyando, and lake locations.

### 2.3. Sample Collection and Processing

**Figure 2:**
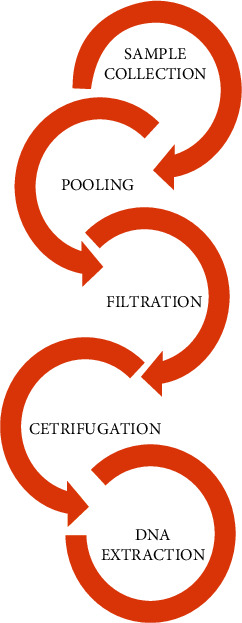
Work flow chart diagram showing sample collection and processing.

Sediment and water samples were purposively collected from fifteen different sites (coordinates and locations: Supplementary material (available here)) of the Winam Gulf Kisumu (Figure 1). Purposive sampling was preferred to obtain a wide range of organic and inorganic pollutants of interest. Water samples were collected in sterile plastic bottles (500 ml), sealed, and transported to the laboratory on cooler boxes (4°C) within 12 hours and stored at −80°C for metagenomics experiments [25]. Sediment samples were collected by scooping a 0–2 cm layer of each and placing it in sterile bottles according to [26]. Eighty-nine samples of water and a similar number of sediments were collected, cleaned with nitric acid, rinsed in distilled water, and transported to the laboratory for processing. A homogenous sample of water and sediment from all the sampling sites, totaling 130 samples, was pooled together and thoroughly mixed. The mixture was sieved using grade 1 filter papers (Whatman ™) to remove large particles and dirt. 10 ml of a 20-liter sample was taken and centrifuged followed by decantation of the supernatant. The experiment was repeated for the entire homogenous sample. The sediment cell debris was vortexed and placed into 2 ml Eppendorf tubes and further centrifuged for 10 minutes at 5000 × *g* for 10 minutes to obtain the pellets for DNA extraction and metagenomics sequencing [27].  Figure 2 summarizes the steps followed.

### 2.4. Physicochemical Analyses of the Sampling Sites

Salinity, total dissolved solids, pH, temperature conductivity, total dissolved solids, and dissolved oxygen were all measured according to [28]. Prior to taking the samples, the temperature of the water was measured with a thermometer, and the values were recorded. The pH meter was calibrated using standard buffers of 4.0, 7.0, and 10.0 to ensure its accuracy before being used to calculate the pH levels. After obtaining 4 ml of each sample, individual readings were recoded and the rod cleansed before taking subsequent pH reading. A conductivity meter was used to test conductivity, which determines how well a solution conducts electricity. To test the electrical conductivity, the conductivity probe was dipped directly into the water samples after the meter had been calibrated using a standard solution, and the results recoded. Using a refractometer, salinity was calculated by first determining the refractive index of a small water sample placed on the prism of the instrument. The refractive index was then translated to salinity using a conversion table that had been constructed. A water sample was put in a cuvette, and a light beam was transmitted through it to measure the turbidity using a turbidimeter. The device determined the turbidity value by counting the quantity of light reflected off the suspended particles in the samples.

Prior to weighing the residual solids, 5 ml of the water samples were evaporated in a preweighed container to measure the total dissolved solids (TDS). The samples were heated to evaporate the water, and then the container was weighed again to determine the TDS concentration. To determine TDS, the volume of the original sample was divided by the weight of the dried solids, and the results were expressed in milligrams per liter. Dissolved oxygen levels were measured using a dissolved oxygen meter. The probe was immersed directly into the water samples, and the instrument measured the partial pressure of oxygen dissolved in the solution. The readings were recorded as dissolved oxygen concentration.

### 2.5. Genomic DNA Extraction and Shotgun Metagenomic Sequencing

DNA extraction was accomplished by modifying the protocol according to [22]. The sample was placed in 2 ml Eppendorf tubes, followed by the addition of 2% of 0.7 ml of extraction CTAB buffer (20 mM EDTA, 0.1 M Tris-HCl pH 8.0, 1.4 M NaCl, and 2% CTAB). 0.4% beta-mercaptoethanol was added right away and incubated at 65°C for 45 min while gently mixing by inversion after every 15 min. The mixture was added with 0.6 ml chloroform-isoamyl in a ratio (24 : 1) and gently mixed for 1 min followed by centrifugation for 10 min at 16000 × *g* and procedure repeated twice. 0.7 ml of cold isopropanol (-20°C) was added to the mixture, and the mixture was gently mixed by inversion. The solution was then centrifuged at 16000 × *g* for 10 min. The extracted DNA was washed twice with 1 ml of 70% ethanol to eliminate salt residues and set to dry overnight with the tubes inverted over filter paper at room temperature. Pellet was then resuspended in 100 ml of TE buffer (10 mM Tris-HCl pH 8.0, and 1 mM EDTA pH 8.0) and stored at -20°C for shotgun metagenomics. Agarose gel electrophoresis was used to check the quality and integrity of the DNA sample, while a Qubit 2.0 Fluorimeter (ThermoFisher Scientific) was used for quantitation of DNA concentration. Shotgun metagenomics analysis is a potential tool in environmental research for identifying microbial assemblages at a specific location and might be helpful in understanding the interaction and taxonomic categories between microbes. The sequencing of the DNA sample was performed at Novogene ((UK) Company Ltd). Genomic DNA fragmentation, end repair and A-tailing, adapter ligation, and PCR amplification were done. The quantified libraries were pooled and sequenced using the Illumina platform.

### 2.6. Metagenomics Data Analysis

Taxonomical abundance was determined by comparing metagenomics reads to a database of taxonomically informative gene families (MicroNR database). Gene prediction was done by MetaGeneMark based on the scaftigs length, and the gene catalogue for each sample was obtained through CD-HIT by keeping the clustering threshold at 95%. Specie annotation was done using DIAMOND software (V0.9.9.110) for alignment of unigenes sequences with those of bacteria, fungi, archaea, and viruses extracted from NCBI's NR database. Functional annotation was inferred based on its similarity to the sequence in the databases (KEGG, eggNOG, and CAZy), while functional category hit distribution was annotated using MG-RAST Subsystems classification.

## 3. Results

### 3.1. Physicochemical Analysis

The physicochemical and statistical results in this study were based on eighty-nine water samples from lake water, River Nyando, River Nyamasaria, River Kisat, and River Wigwa.

The values pH, salinity, TDS, EC and COD were within acceptable WHO standards, whereas turbidity was above the WHO acceptable standards (Table 1).

### 3.2. Taxonomic Classification of Microbial Communities in Winam Gulf

**Figure 3:**
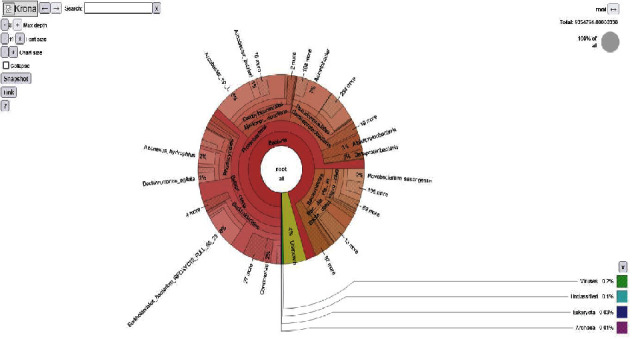
Krona chart showing the taxonomic hierarchy and percentage composition of the microbial community including phyla, class, family, order, and species. It is evidence that relative abundance is high in *Bacteria*, *Proteobacteria*, and *Betaproteobacteria*, and antibiotic-resistant species were identified in the *Bukholodriales bacterium.*

**Figure 4:**
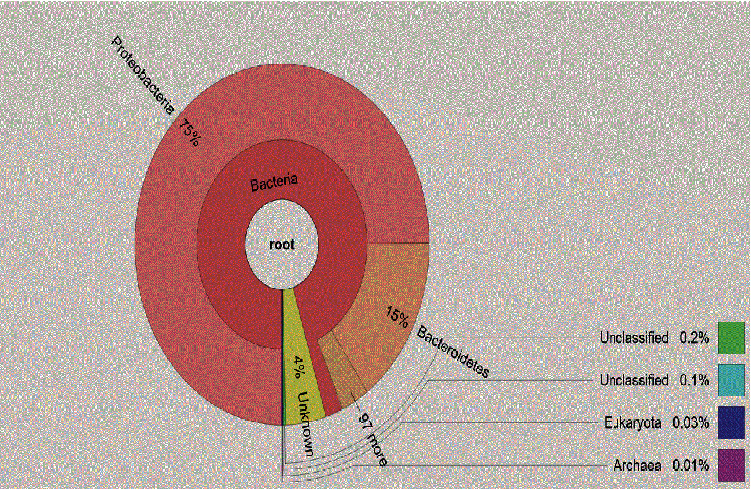
Relative abundance of bacteria annotated as taxa in samples collected from Winam Gulf showing the distribution percentages of the bacterial phylum composition in the total sample. The highest relative abundance was bacteria with *Proteobacteria* depicting the highest phylum and *Verrucomicrobia.*

Krona analysis revealed the diversity of microbial communities in Winam Gulf (Figure 3). These results represent pooled samples from different sampling sites. *Bacteria* were highly abundant at 95%, while *Eukaryotes* and *Archaea* were 0.03% and 0.01%, respectively. The unclassified microbes were 0.3%, while the unknown microorganism were 4%. A relative abundance of annotated taxa of the bacteria phylum with *Proteobacteria* being highly enriched at 75%, *Bacteriodes* being enriched at 15%, and *Verrucomicrobia* being least enriched at 2% (Figure 4).

**Figure 5:**
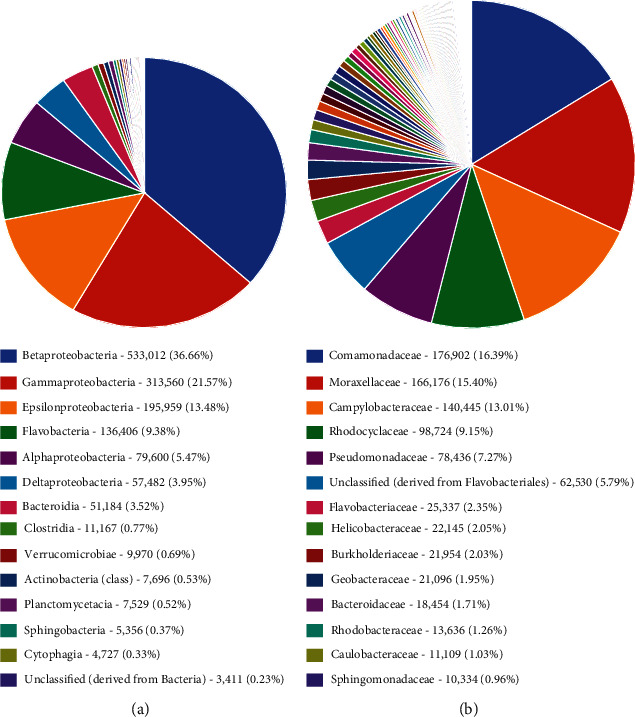
(a) Frequencies of the bacteria taxa class showing the percentages of the bacteria in the sample from Winam Gulf. (b) Frequencies of the bacteria taxa family showing the percentages of the bacteria in the sample from Winam Gulf.

Class taxonomic classification was reported as follows: *Betaproteobacteria (*36.7*%)* was the most abundant class, followed by *Gammaproteobacteria* (21.6%), *Epsilonproteobacteria* (13.5%), *Flavobacteria* (9.3), *Aphaproteobacteria* (5.5%), *Deltaproteobacteria* (4%), *Bacterioidia* (3.5%), *Clostridia* (0.8%), *Verrumicrobiae* (0.7%), *Actinobacteria* (0.5%), *Planctomycetacia* (0.5%), *Sphingobacteria* (0.4%), and *Cytophagia* (0.3%). The least was derived from unclassified derived from bacteria (0.23%) (Figure 5(a)). In family taxonomic classification, on the other hand, indicates that *Comamonadaceace* (16.4%) was found to be the most abundant, followed by *Moraxellaceae* (15.4%), *CampylobacteraceaeI* (13%), *Rhodocyciaceae* (9.2%), *Pseudomonadaceae* (7.3%), unclassified derived from *Flavobacteriales* (5.8%), *Flavobacteriaceae* (2.4%), *Helicobacteriaceae* (2.1%), *Bulkholderiaceae* (2.%), *Geobacteriodaceace* (*2*%), *Bacteriodaceae* (1.7%), *Rhodobacteraceae* (1.3%), *Caulobacteraceae* (1%), and the least abundance was *Sphingomonadaceae* (1%) (Figure 5(b)).

**Figure 6:**
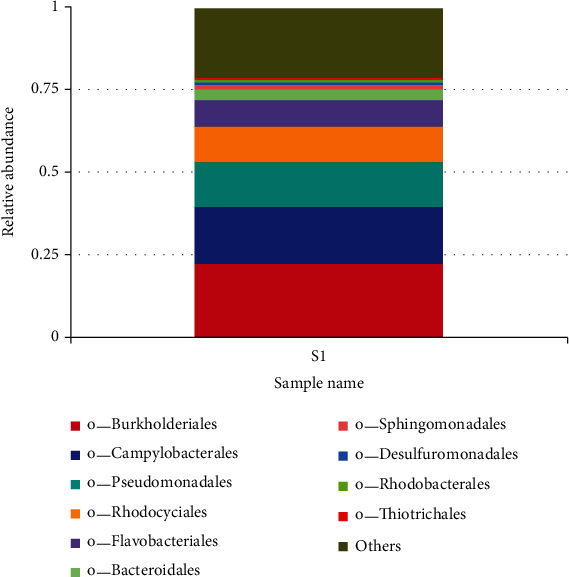
Relative abundance of bacteria taxa at order level. The *x*-axis represents the sample name l, and the *y*-axis represents the relative abundance.

In the order of taxonomic classification, the most relative abundance order was *Burkholderiales* (24.7%) and followed by *Pseudomanadales* (19.2%), *Campylobacterales* (13.8%), *Rhodocyciales* (7.7%), *Bacteroidales* (4%), *Sphingomonadales* (1.7%), *Desulfuromonadales* (1.1%), and *Rhodobacterales* (1.30%), and the least abundance was *Thiotrichales* (1. %) (Figure 6).

**Figure 7:**
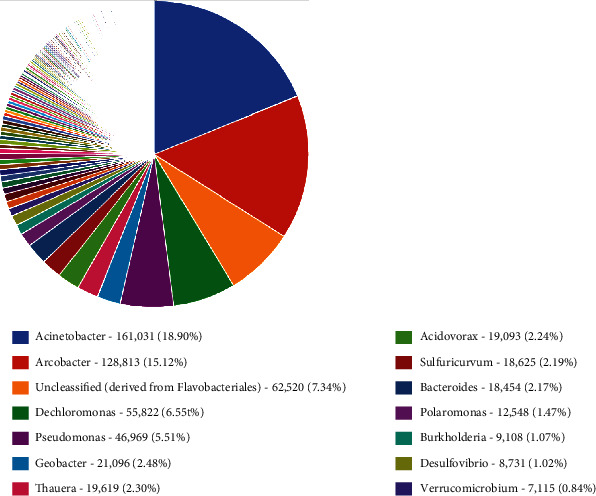
Relative abundance of the bacteria taxa at genus level; taxonomic category based on shotgun metagenomics datasets showing the percentages of the bacteria in the sample from Winam Gulf, Kisumu.

In genus taxonomic classification, the most relative abundance was genus *Acinetobacter* (18.9%), followed by *Arcobacter* (14.1%), unclassified derived from *Flavobacteriales* (7.3%), *Dechloromonas* (6.6%), *Pseudomonas* (5.5%), *Geobacter* (2.5%), *Thauera* (2.3%), *Acidovorax* (2.2%), *Sulfuricurvum* (2.2%), *Bacteroides* (2.2%), *Polaromonas* (1.5%), *Burkholderia* (1.1%), and *Desulfovibrio* (1.), and the least abundance was *Verrumicrobium* (0.8%) (Figure 7).

**Figure 8:**
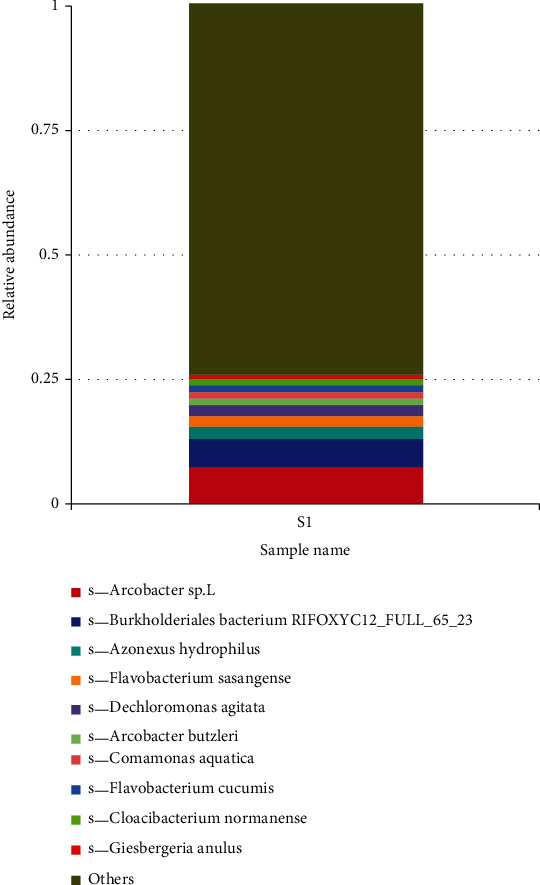
Relative abundance of bacteria taxa at species level; taxonomic category based on shotgun metagenomics datasets showing the percentages of bacteria in the sample from Winam Gulf, Kisumu.

In species taxonomic classification, the relative abundance of bacteria at species level was genus *Arcobacter (*6%), *Burkholderiales bacterium RIFOXYC12-FULL-65-23 (*6%), *Azonexus hydrophilus* (2%), *Flavobacterium sasangense* (2%), *Dechloromonas agitata* (2%), *Arcobacter butzleri* (1%), *Comamonas aquatic* (1%), *Flavobacterium cucumis (*1%), and *Cloacibacterium normanemse* (1%), and the least abundance was *Giesbergeria annulus* (1%) (Figure 8).

### 3.3. Functional Annotation

**Figure 9:**
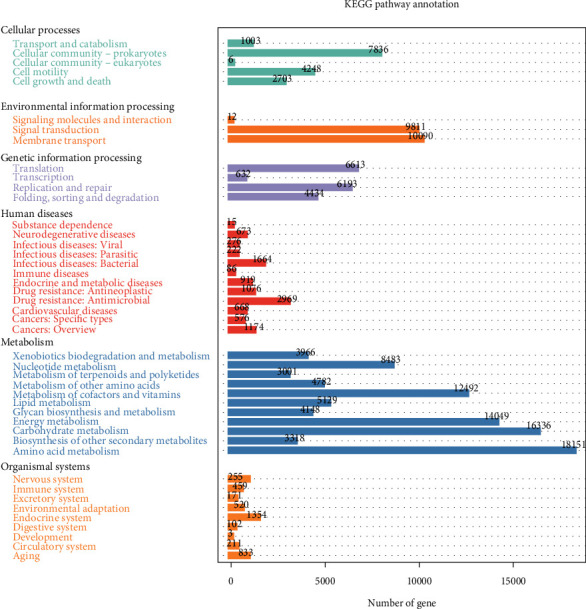
Relative abundance of the KEGG-level functional categories based on shotgun metagenomics data set for freshwaters and sediments from Winam Gulf. The highest relative abundance being the metabolism and the lowest organismal systems.

**Figure 10:**
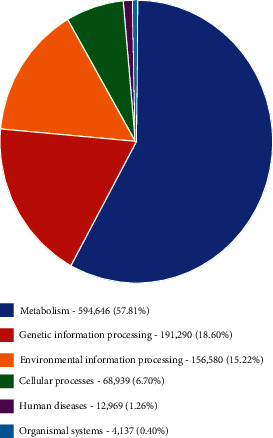
Relative abundance of the KEGG-level functional categories based on shotgun metagenomic dataset for freshwaters and sediments showing the percentages and genes. Metabolism being the highest relative abundance and organismal the least.

According to functional abundance, various analyses were performed to predict functional groups of operating taxonomic units based on the KEGG database. The results indicated metabolism activities at 57.8% ( 594,646 genes) with the highest abundance of genes, followed by genetic information processes at 18.6% (191,290 genes). Environmental information processes at 15.22% (156.580 genes) and cellular processes at 6.7% (68,939 genes). The human diseases were at 1.26% (12969 genes), and the organismal system was at 0.40% (4,147 genes) (Figures 9 and 10).

### 3.4. KEGG Pathway

**Figure 11:**
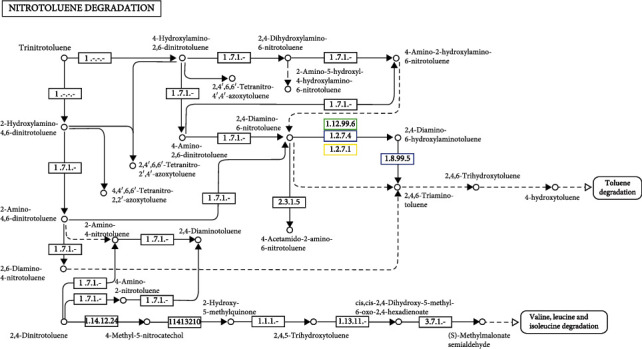
Nutrotuolene degradation pathway showing 1.12.99.6 hydrogenase large subunit biodegradation by microbes. 1.8.99.5 dissimilatory sulfite reductase alpha subunits are responsible for biodegrading nutrotuolene and finally degrading tuolene and amino acids, namely, valine, leucine, and isoleucine.

KEGG pathway analysis revealed that metabolism had the highest percentage in the study; we highlighted the few pathways of importance to bioremediation. The genes involved in the nitrotoluene breakdown process were uncovered by metagenomics analysis. Leucine, valine, isoleucine, and tuolene, which are safe for the environment, were the final organic molecules to degrade, as shown by the pathway (Figure 11).

**Figure 12:**
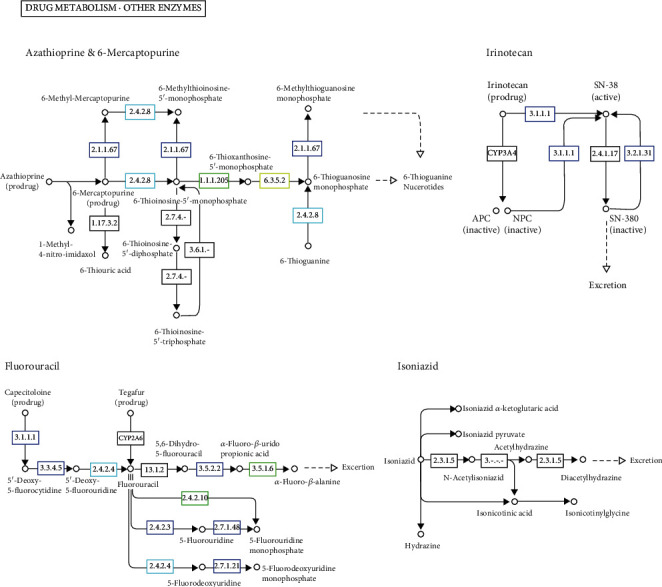
Drug and enzyme metabolism pathways show microbial genes like 3.4.2.8 hypoxanthine phosphoribosyl transferases responsible for drug metabolism and degradation of azathioprine, 6-thiguanine, and 5-triphosphate inactivating drug ingredients. 3.1.1.1 carboxylestrase keg orthogus genome is responsible for drug metabolism and biodegradation of irinotecam to 3.2.1.31 beta-glucuronide which is responsible for its biodegradation, thus inactivating the drug-active ingredients. 2.7.1.21 thymidine kinase is responsible for xenobiotic biodegradation and drug metabolism.

The KEGG analysis identified the genes crucial to the drug's metabolism. The medications that were broken down were isoniazid, azathioprine and mercaptopurine, irinotecan, and fluorouracil. The process by which bacteria remove active drug compounds from the aquatic habitat is shown (Figure 12).

**Figure 13:**
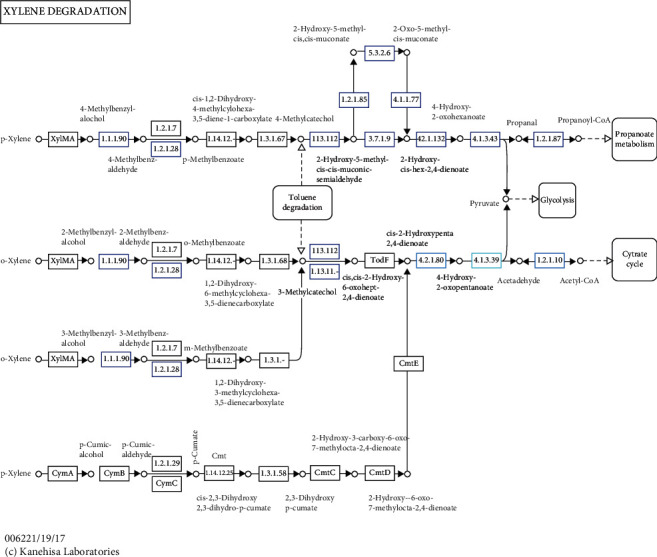
Xylene degradation pathway1.1.1.90 aryl-alcohol dehydrogenase xylene degradation by microbes in diverse environment. 1.2.187 propanol hydrogenase forms a bifunction complex with E.4.1.3.43 4-hydroxy-2-oxhexanoate aldoses with a tight channel connecting two subunits (1, 2, and 3). Also, act more slowly on glycol aldehyde and butanol. In pseudomonas species, the enzymes form a bi-functional complex with E4.1.3.39, 4-hydroxy-2-oxovalerate aldose. The enzymes from bacteria *Bukholderia*, *xenovers*, and *Thermus thermophillus* also perform the reaction of EC 1.2.2.1.10; acetaldehyde dehydrogenase (acetylating) NADP^+^ can replace NAD^+^ with a much lower rate, and the products are propanoate metabolism and citrate cycle.

Metagenomic analysis was used to identify the genes involved in the process that breaks down xylene. The hazardous xylene is degraded, as shown in the route below (Figure 13). The cytrate cycle and propanoate metabolism were the last phases.

### 3.5. Carbohydrate Metabolism

**Figure 14:**
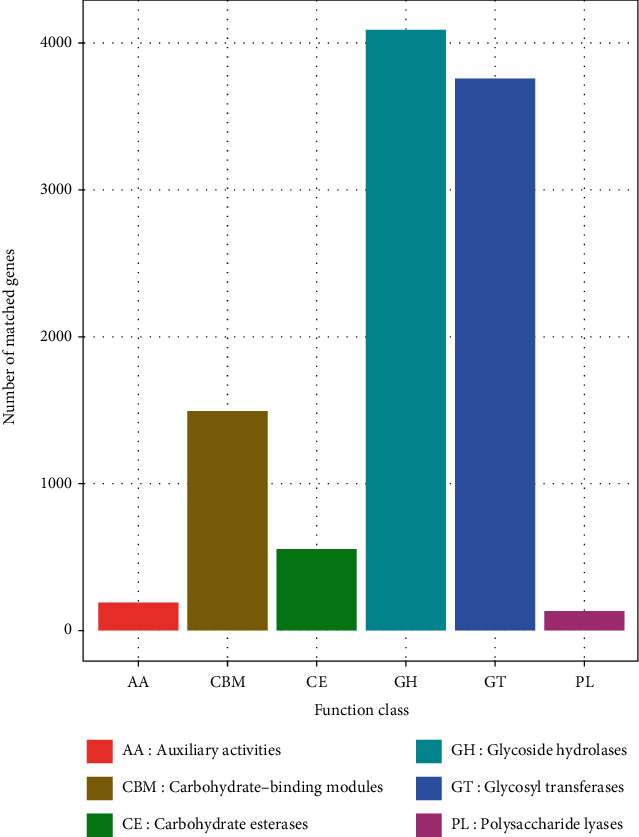
Predicted functional groups of operating taxonomic units based on CAZy enzymes based on shotgun metagenomics data set for freshwater and sediments. The glycoside hydrolases and glycosyl transferases were among the highest relative abundance genes.

In the determination of carbohydrate enzyme (Figure 14), glycoside hydrolases displayed the highest number of matched genes (≥4000), followed by glycosyl transferases with 3800 number of matched genes. Carbohydrate-binding modules matched 1500 genes, while Carbohydrate esterases matched approximately 600 genes. Auxiliary activities and polysaccharide lyases matched genes less than 500.

### 3.6. eggNOG Functional Classification

**Figure 15:**
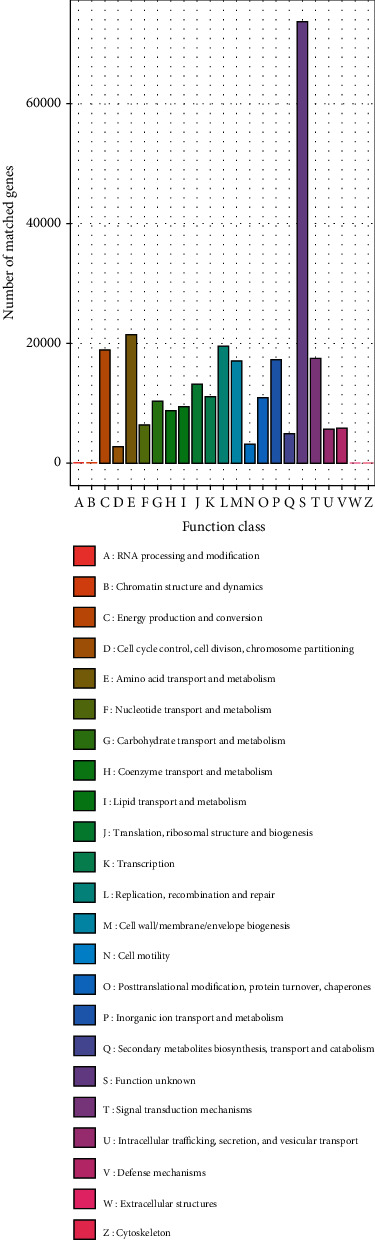
Relative abundance of eggNOG and orthologous groups (OGs) of proteins at different taxonomic levels; functional categories based on shotgun metagenomics database for freshwater and sediments. Depicting functional unknown highest genes and RNA processing and cytoskeleton among the lowest genes.

In the determination of orthologous groups (OGs) of proteins (Figure 15), the highest relative abundance was the genes for unknown functions with above 70,000 matched genes, followed by amino acid and derivatives and metabolism; replication, recombination, and repair; energy production; and conversion, while among the least was RNA processing and modification, chromatin structure and dynamics, and the cytoskeleton.

### 3.7. MG-RAST Functional Classification

**Figure 16:**
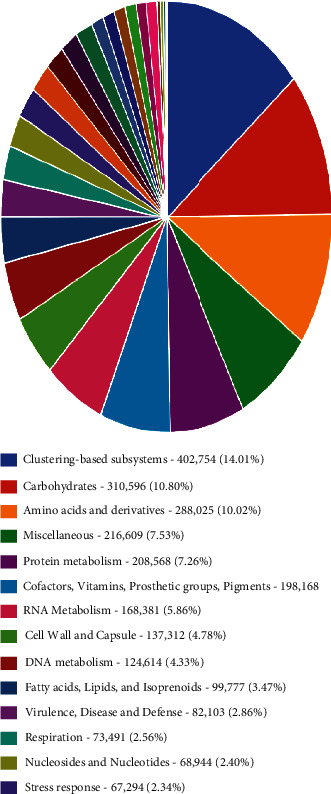
Relative abundance of MG-RAST functional category hits distribution annotated subsystem classification based on shotgun metagenomics data set for freshwater and sediments.

Functional category hit distribution annotated subsystems classification (Figure 16). Most abundant was clustering–based subsystems with 14.1%, followed by carbohydrates at 10.8%, amino acids at 10.02%, miscellaneous at 7.5%, protein metabolism at 7.3%, cofactors vitamins, prosthetic groups, pigments (%), RNA metabolism at 5.86%, cell wall and capsule at 4.78%, DNA metabolism at 4.3, fatty acids, lipid and isoprenoid at 3.5%, virulence, disease and defense at 2.9%, respiration 2.6%, and nucleosides and nucleotides at 2.4%, and the least abundant was the stress response at 2.3%.

## 4. Discussion

Shotgun metagenomic analysis is a potential tool in environmental research for identifying microbial assemblages at a specific location and might be helpful in understanding the interaction and taxonomic categorization between microbes. Microbial assemblages, which are essentially bacteria in the water system, play a significant role in the biogeochemical processes that support the aquatic ecosystem. The high levels of diversity in these communities contribute to their functionality and stability. However, the abundance of microbial communities is significantly impacted by the presence of multiclass pollutants and other ecological factors [29]. The current study used shotgun metagenomic analyses to provide a taxonomic assessment and functional diversity of microbes in the polluted aquatic ecosystem of Winam Gulf of Lake Victoria, Kenya. Previous studies have indicated high levels of pollution in the aquatic ecosystem of Lake Victoria [6, 30, 31]. The presence of pharmaceutical residues, heavy metals, personal care products, agrochemicals, antiretrovirals, and drugs of abuse is known to alter microbial assemblages in aquatic environments with calamitous consequences to human health [14]. Analysis of the physical chemical properties of the sampling sites showed that pH ranged from 6.5 to 8.5 which is often considered an ideal optimal health of freshwater ecosystems and suitable for the survival of most freshwater organisms [6]. This pH is favorable for the presence of diverse plants and animals and for natural breakdown of organic matter and nutrient cycling [28]. This is consistent with the findings of a previous study on Lake Victoria [32]. The values of pH, salinity, TDS, EC, and COD were within acceptable WHO standards, whereas turbidity was above the WHO acceptable standards, consistent with previous studies [33].

The findings of this study indicate that the relative sequence abundance of the bacterial assemblages represented was the most dominant, as presented by the Krona charts, pie chart, and bar graphs. (figure/plates). The bacterial structure was further analyzed into relative abundance of bacteria phyla, and *Proteobacteria* was found to be the most abundant. This study is consistent with those reported by previous studies [23]. *Bacteriodes* was the second most dominant phylum, and the least relative abundance was the *Verrucomicrobia* at 2%. These results are consistent with those reported in previous studies, where *Proteobacteria* and *Bacteriodes* were the dominant phyla, and *Verrucomicrobia* was among the least phyla reported [34]. The dominant relative abundance of *Proteobacteria* may imply pollution associated with hydrocarbons in water sediments [35]. *Bacteroidetes* are naturally found in the human gut; therefore, their presence in the freshwater signifies sewage pollution [36]. The presence of *Bacteroidetes* are organism of importance, as they have been reported to have carcinogenic effects on human and animals, such findings were reported in a study [37]. We hypothesize that the numerous cancer cases that have been reported in the region near the Lake Victoria are probably due to the pollution from the Winam Gulf [9]. It is important for the scientific community to look into the potential risks posed by these pollutants to fish and human health because it is noted that the local community consumes and exports fish; the pollutants enter the food down the food chain downstream and are likely to have an impact on the local community and those who import fish from Lake Victoria. *Verrucomicrobia* are naturally freshwater habitats, but they are in lower percentages, proving pollution alters the microbial community structures [38].

The bacteria communities were further categorized into class, and the relative abundance dominant class was the *Betaproteobactetria*, followed by *Gammaproteobacteria*, *Epsilonproteobacteria*, *Flavobacteriia*, *Alphaproteobacteria*, *Deltaproteobacteria*, *Bacterioidia*, *Clostridia*, *Verrumicrobiae*, *Actinobacteria*, *Planctomycetacia*, *Sphingobacteria*, *Cytophagia* and unclassified derived from bacteria, respectively. *Betaproteobacteria* have a wide range of habitats from the natural waters to human pathogens, so their presence may signify pollution [39]. *Epsilonproteobacteria* are known to be autotrophic and play an important role in Co_2_ fixation in aquatic ecosystem [40]. *Epsilonproteobacteria* have been known to be prevalent in animals and human digestive tracts, serving as pathogens or symbionts; their energy metabolism involves hydrogen, oxidizing reduced sulfur or formate, and coupling with the oxygen or nitrate reduction. *Gammaproteobacteria* are widely distributed and abundant in various ecosystems such as soils, freshwater lakes, and rivers [41].The phylum *Bacteroidetes* is diverse which includes *Flexibacter*, *Cytophaga*, and *Bacteroides* [42, 43]. The *Bacteroidetes* phylum is made up of four classes: *Flavobacteria, Bacteriode, Sphingobacteria*, and Cytophaga, with over 7,000 diverse species [44]. The *Bacteroidetes* phylum found in the Winam Gulf included the *Flavobacteria, Bacteriodiia* unassigned, and unclassified *Bacteriodetes*. *Flavobacteria* which is made up of many aquatic species). *Falvobacteria* contains species of opportunistic human pathogens and antimicrobial-resistant genes [44]. *Bacteroidetes* species have economic impacts on freshwater fish, which cause infections that may have negative effects on wild and farmed fish [45, 46]. *Bacterioidia*, which forms the dominant of animal microbiota especially those found in the gastrointestinal tract, are pathogens and frequently found in freshwater, oceans, and soils [47]. The findings of the relative abundance family were as follows: *Commamonadaceae*, followed by *Moraxellaceae*, then *Campilobacteriaceae*, *Rhodocyliaceae*, *Pseudomonaceae*, *Flavobacteriaceae*, among others. *Campylobacteriaceae* are natural habitat of birds and warm-blooded animals and thus may find their way to freshwater systems through pollution. *Commamonadaceae*, Pseudomonaceae, have natural habitat in terrestrial and aquatic ecosystems but make a major group of human pathogens and are found in water systems through fecal contamination [48]. The existence of these channels both validates the cause and provides the public health agency with information on effective treatments. Pathogenic bacteria, particularly disease-causing pathogens present in water, have consistently been linked to cholera instances among people in Lake Victoria [49]. This category has been documented, and it follows that this study could suggest a further study to interrogate the unclassified. *Burkhoideriales* were the most abundant order; it naturally occurs in soils and water and is known to infect both humans and animals by spreading zoonotic illnesses and respiratory disorders through contaminated water [50]. *Burkholderiales* are also known to possess antibiotic-resistant genes and not easy to remove from the environment [51]. *Rhodocyciales* is an abundant bacterium order found in wastewater treatment plants and plays the function of denitrifying [52]. *Pseudomonas* is known to inhabit soils, water, and vegetation but is also pathogenic to human health [53]. In the samples from the aquatic ecosystem of the Winam Gulf in Kisumu, taxonomic diversity reveals the existence of antibiotic-resistant bacteria. The unclassified group of *Burkholderiales* and *Burkholderiales bacterium* shows resistance to antibiotics. These groups represent a higher percentage of antibiotic resistance, thus posing risk to public health. Studies have shown *Acinetobacter* is moderately to highly resistant to several groups of antibiotics such as fluoroquinolones, aminoglycoside, tetracycline, and other classes of antibiotics [54]. *Pseudomonas* spp. has been proven to possess a high level of intrinsic resistance to antibiotic through restricted outer membrane permeability, thus pumping antibiotic out of the cell and producing enzymes such as *ß*-lactamase [55]. *Sulfuricurvum* which are minority sulfur-metabolizing bacteria [56].

Studies on metagenomics analysis have highlighted the importance of functional annotation for microbial community diversity. KEGG pathways, CAZy, eggNOG, and the MG-RAST analysis were the four functional analyses carried out in this work. The relationship between the metabolic networks and genomic networks, as well as how the encoded genes for biochemical reactions, have been demonstrated using the KEGG pathway [57]. In the above findings, the most abundant KEGG functional categories were metabolism, gene information, environmental formation process, human diseases, and organismal systems. The genes that are responsible for metabolisms were higher which was also observed in a previous study [58]. Microbes have to biosynthesize substrate and products to get energy for survival [59]. Genetic information processing was second dominant genetic information systems of microbes which are responsible for gene transcription, translation, replication, folding sorting, and degeneration [60]. Environmental information processing genes were the third predicted by the KEGG pathway based on shotgun metagenomics. Environmental information processing is a key due to the interaction of organisms and the environment for a period of time, resulting in evolution and diversity [61]. The fourth functional genes were the cellular processes of the microbes which are important in carrying out specialized functions like providing the body structure, nutrients uptake from food, and converting them to energy [62]. The KEGG pathway and annotation show the genes for human diseases ranged from cancers, metabolic disease, cardiovascular disease, endocrine substance dependence, and drug resistance which may have been triggered by the presence of pollutants. They imbibe disease-causing genes through water, aquatic plants, and animals, causing a lot of health risks in the community using the water systems at the Winam Gulf ecosystem [63]. Xylene, drug and enzyme metabolism, and nutrotolene biodegradation were significant KEGG analytical pathway. Unsafe for the environment, xylene is a cyclic hydrocarbon that is utilized as a solvent in dye, paint, medical technology, and other sectors. In both humans and animals, xylene has negative effects on the respiratory, neurological, cardiovascular, and renal systems [64]. The metabolic pathway demonstrates the presence of genes that can be explored in the biodegradation and bioremediation of this harmful chemical. Nutrotuolene from a manufacturing plant's waste water has been dispersed into the atmosphere and surface water [65]. Public health concerns from nitrotoluene include breathing difficulties, skin and eye irritation, coughing, a rapid heartbeat, nausea, vomiting, convulsions, and even death. By employing the local bacteria, the bioremediation processes provide a remedy by eliminating the dangerous chemical from the environment. Previous studies have demonstrated pharmaceutical contamination in Lake Victoria [66]. Drugs like isoniazid that are used to treat tuberculosis are found in water, which is a public health problem as the world fights TB treatment resistance [67]. These xenobiotics may be removed from aquatic ecosystems by this mechanism, which could be a useful tool.

The CAZyme pathway has been used to encode carbohydrate enzymes in the microbial genome, thus having an elaborate enzyme mechanism to utilize complex carbohydrates from different sources [68]. The most relative abundance genes in CAZyme were the GH, followed by GT, then CBM, and then CE, and AA and Pl were the least abundance. The above results were found to agree with previous studies [68, 69]. The GH comprises a large group of enzymes responsible for polysaccharide metabolism such as chitin, cellulose, xylan and starch [70]. GT enzymes play a key role in carbohydrates biosynthesis which is important synthesis of saccharides and glycosylation of molecules in aquatic environment [71]. The CAZy relative abundance results at the Winam Gulf suggest that enzymes responsible in carbohydrates metabolism can be influenced by the adaptation of microbial profiles in specific community structures [72]. The other functional annotation carried out was the MG-RAST subsystem classification and these looks at the sets of proteins that share the protein coding regions from the assembled contigs. Clustering-based subsystems were the largest category, followed by the carbohydrates, amino acids and derivatives miscellaneous, protein metabolism, cofactors, vitamins, prosthetic groups, pigments, RNA metabolism, cell wall and capsule, DNA metabolism, fatty acid lipids and isoprenoid virulence disease, and defense respiration nucleosides and nucleotides, and the least abundant was the stress response. The presence of the least relative abundance of the stress response enzymes signifies that the microbial community structures at the Winam Gulf have adapted to the multiclass polluted ecosystem. The clustering–based subsystem that couples with functional evidence among proteins, but its exact role on metabolic pathway is not yet established [73]. In this study, nonsupervised orthologous functional annotation, amino acid sequences of predicted genes were aligned with the eggNOG through the BLAST database. In this study, a total of twenty-six eggNOG functional categories were observed through the functional analysis. The metagenomics findings involved in amino acid transport and metabolism, replication, recombination, and repair; energy production and conversion; cell wall/membrane/envelope biogenesis; inorganic ion transport and metabolism; signal transduction and mechanism; and lastly, translation ribosomal structure and biogenesis. This suggests that the majority of the functional activities in the Winam Gulf microbiome collected were involved in replication, growth, response to changes in the environment, and metabolism. The presence of signal transduction and mechanism among the dominant genes may suggest pollution; thus, organisms have adapted to changing environmental conditions [74]. This category of metagenome analysis revealed a gap that exists in the functions of large communities of microorganisms, thus creating a chance of discovering new activities in functional library screening.

## 5. Conclusions

This study revealed, for the first time ever, rare genes important in medical, industrial, and environmental microbiology, which is essential for future investigations of microbial biogeochemical connections in ecosystems. In Kenya, inadequate water supply is still a significant issue. The Winam Gulf of Lake Victoria, Kenya, has been examined for its microbial profiles, which highlight novel microorganisms of public health interest. The relative abundance and collective richness of enzymes have a collective ecological purpose in the environment. A specific tactic employed by microbial assemblages to deal with the metabolism of organic carbon in aquatic environments is documented. This study documents genes for many hydrolases and lipases and two enzymes with potential biotechnological applications. Metabolic pathway footprints related to different primary nutritional groups are reported. The findings of the current study highlights fundamental aspects influencing water microbiomes in the aquatic ecosystem of Winam Gulf of Lake Victoria, Kenya, which make it possible to design an appropriate bioremediation strategy and develop an environmental management system geared towards restoration of the Winam Gulf ecosystem.

## Figures and Tables

**Table 1:** Mean and standard values of physicochemical parameters of water in Winam Gulf of Lake Victoria and WHO standards.

	*N*	pH	Salinity	Turbidity	TDS	EC	COD
Kiwasco water	89	8.50 ± 0.23	0.11 ± 0.04	6.93 ± 2.02	184.42 ± 2.74	368.45 ± 11.60	520.60 ± 39.40
Nyando water	89	7.06 ± 0.05	0.10 ± 0.01	7.29 ± 1.29	182.87 ± 7.02	365.21 ± 3.27	381.50 ± 4.92
Kisat water	89	7.04 ± 0.09	0.04 ± 0.02	6.67 ± 1.28	299.20 ± 1.36	604.00 ± 4.17	272.33 ± 52.54
Lake water	89	7.07 ± 0.38	0.02 ± 0.01	7.12 ± 0.17	41.9 ± 1.46	84.43 ± 0.67	321.00 ± 6.76
Wigwa water	89	7.20 ± 0.12	0.05 ± 0.03	9.74 ± 1.44	284.13 ± 3.18	588.47 ± 5.49	284.00 ± 7.74
Nyamasaria water	89	7.66 ± 0.07	0.21 ± 0.04	11.06 ± 1.54	266.53 ± 28.66	535.84 ± 11.40	397.25 ± 6.64
WHO STDS		6.5-8.5	>0.5 ppt	>5NTU	>500	>1000S/m	<5 mg/O_2_l

There was significant difference between physicochemical parameters in various water sample from different sites (*F* = (*P* < 0.01).

## Data Availability

The data is available in this manuscript.

## Supplementary Materials

Supplementary Materials The supplementary material consists of a list of coordinates for the 31 sampling sites found in Figure 1, showing their precise location on the earth's surface within the Winam Gulf. The location was done in UTM, consisting of an easting value representing the distance in meters eastward from a central meridian within the zone. The northing value represents the distance in meters north from the equator.
