# Supplementary material for: Shotgun Metagenomic Analyses of Microbial Assemblages in the Aquatic Ecosystem of Winam Gulf of Lake Victoria, Kenya Reveals Multiclass Pollution
Source: Biomed Res Int. 2023 Jul 21;2023:3724531. doi: 10.1155/2023/3724531 (PMC10382247; doi:10.1155/2023/3724531)
Supplement: Supplementary Materials — The supplementary material consists of a list of coordinates for the 31 sampling sites found in Figure 1, showing their precise location on the earth's surface within the Winam Gulf. The location was done in UTM, consisting of an easting value representing the distance in meters eastward from a central meridian within the zone. The northing value represents the distance in meters north from the equator. [file 3724531.f1.docx]

The table below consists of a list of coordinates for the 31 sampling sites found in Figure 1, showing their precise location on the earth's surface within the Winam Gulf. The location was done in UTM, consisting of an easting value representing the distance in meters eastward from a central meridian within the zone. The northing value represents the distance in meters north from the equator.

**Supplementary material showing Coordinates in Universal Transverse**

**Mercator (UTM)**

| **Site Name** | **Easting (m)** | **Northing (m)** | **Location** |
| --- | --- | --- | --- |
| Coord 1 | 695021.44 | 9990768.83 | On Kisat River |
| Coord 2 | 695041.95 | 9990805.53 | On Kisat River |
| Coord 3 | 695292.15 | 9990885.55 | On Kisat River |
| Coord 4 | 697142.34 | 9989179.06 | Stormwater |
| Coord 5 | 695293.26 | 9987900.89 | Stormwater |
| Coord 6 | 694588.07 | 9990444.19 | In Lake Victoria |
| Coord 7 | 695101.12 | 9989950.29 | In Lake Victoria |
| Coord 8 | 697948.19 | 9988145.57 | On Waigwa River |
| Coord 9 | 697495.98 | 9987143.58 | Kiwasco Outlet |
| Coord 10 | 699004.77 | 9987553.38 | Stormwater |
| Coord 11 | 701749.26 | 9986846.43 | Stormwater |
| Coord 12 | 713574.33 | 9980978.86 | On Nyando River |
| Coord 13 | 713691.33 | 9980855.95 | On Nyando River |
| Coord 14 | 707222.92 | 9966758.02 | On Nyando River |
| Coord 15 | 693520.54 | 9983875.81 | On Nyamasaria River |
| Coord 16 | 694262.00 | 9990245.00 | In Lake Victoria |
| Coord 17 | 694490.00 | 9989777.00 | In Lake Victoria |
| Coord 18 | 693660.00 | 9989999.00 | In Lake Victoria |
| Coord 19 | 694399.00 | 9989296.00 | In Lake Victoria |
| Coord 20 | 694953.00 | 9989745.00 | In Lake Victoria |
| Coord 21 | 693878.00 | 9989606.00 | In Lake Victoria |
| Coord 22 | 694154.00 | 9988933.00 | In Lake Victoria |
| Coord 23 | 693338.00 | 9989711.00 | In Lake Victoria |
| Coord 24 | 693415.00 | 9989258.00 | In Lake Victoria |
| Coord 25 | 693677.00 | 9988651.00 | In Lake Victoria |
| Coord 26 | 693367.00 | 9988909.00 | In Lake Victoria |
| Coord 27 | 693072.00 | 9989401.00 | In Lake Victoria |
| Coord 28 | 693885.00 | 9989355.00 | In Lake Victoria |
| Coord 29 | 693367.00 | 9988909.00 | In Lake Victoria |
| Coord 30 | 693004.00 | 9988677.00 | In Lake Victoria |
| Coord 31 | 693499.00 | 9988294.00 | In Lake Victoria |
